# Exploring Hydrothermal Synthesis of SAPO-18 under High Hydrostatic Pressure

**DOI:** 10.3390/nano12030396

**Published:** 2022-01-26

**Authors:** Raquel Simancas, Masamori Takemura, Yasuo Yonezawa, Sohei Sukenaga, Mariko Ando, Hiroyuki Shibata, Anand Chokkalingam, Kenta Iyoki, Tatsuya Okubo, Toru Wakihara

**Affiliations:** 1 Institute of Engineering Innovation, The University of Tokyo, Tokyo 113-8656, Japan; rsimancas@chemsys.t.u-tokyo.ac.jp (R.S.); yone-rice@chemsys.t.u-tokyo.ac.jp (Y.Y.); anand@chemsys.t.u-tokyo.ac.jp (A.C.); 2 Department of Chemical System Engineering, The University of Tokyo, Tokyo 113-8656, Japan; takem003@chemsys.t.u-tokyo.ac.jp (M.T.); k_iyoki@chemsys.t.u-tokyo.ac.jp (K.I.); okubo@chemsys.t.u-tokyo.ac.jp (T.O.); 3 Institute of Multidisciplinary Research for Advanced Materials, Tohoku University, Sendai 980-8577, Japan; sohei.sukenaga.d3@tohoku.ac.jp (S.S.); hiroyuki.shibata.e8@tohoku.ac.jp (H.S.); 4 Graduate School of Engineering, Tohoku University, Sendai 980-8579, Japan; mariko.ando.b2@tohoku.ac.jp

**Keywords:** silicoaluminophosphates, hydrothermal synthesis, high pressure, water content, non-conventional synthesis

## Abstract

The effect of external hydrostatic pressure on the hydrothermal synthesis of the microporous silicoaluminophosphate SAPO-18 has been explored. The crystallization of the SAPO-18 phase is inhibited at 150 °C under high pressures (200 MPa) when using relatively diluted synthesis mixtures. On the contrary, the use of concentrated synthesis mixtures allowed SAPO-18 to be obtained in all the studied conditions. The obtained solids were characterized with XRD, SEM, ICP-AES, TG and ^27^Al and ^31^P MAS NMR spectroscopy. The results highlight the importance of the external pressure effect on the hydrothermal synthesis of molecular sieves and its influence on the interaction between the organic molecule and the silicoaluminophosphate network.

## 1. Introduction

Zeolites and related materials have been used in a wide range of applications as ion exchangers, adsorbents and catalysts [1,2,3]. Aluminophosphate (AlPO) molecular sieves, an analogue of conventional zeolites, are constructed by alternating corner-sharing PO_4_ tetrahedra and Al polyhedral (AlO_4_, AlO_5_ and AlO_6_) by bridging O atoms forming neutral networks. The number of reported structures of this family of microporous materials has been growing since the first reported synthesis by Wilson and Flanigen in 1982 [4] until more than 300 open-framework aluminophosphate structures were published in the literature by 2020 [5]. Many of these aluminophosphate structures are also included in the Database of Zeolite Framework Types approved by the Structure Commission of the International Zeolite Association (IZA-SC) [6]. The substitution of part of the P atoms by Si atoms introduces Brønsted acidity in the materials and expands the catalytic applications of the obtained silicoaluminophosphates (SAPOs) [7,8,9,10].

AlPO and SAPO materials are usually synthesized by the hydrothermal treatment of an aqueous mixture containing organic molecules, such as amines, alkyl ammonium cations and alkanolamines such as Organic Structure-Directing Agents (OSDAs) [9,11,12]. Many parameters control the hydrothermal synthesis of microporous materials [13,14,15,16], however, pressure, another variable to consider, has been long overlooked in the synthesis of these solids that are typically prepared under autogenous pressure.

Although the influence of the external pressure in the hydrothermal crystallization of microporous materials remains unclear, several studies have been performed in order to rationalize its effect. Synthesis pressure plays an important role in the solubility of precursors and supersaturation of the synthesis mixture. In general, the solubility increases with the pressure; however, too high-pressure values could lead to dense phases [17,18]. Several zeolites have been prepared in the absence of OSDA under high external pressure (100 MPa) at relatively high temperatures (170–270 °C) for 60 days [19,20]. In this method, distilled water was added to a glass precursor having the same composition as that of the respective natural zeolite. Under high-pressure, the glass is dissolved in the water until the mixture reaches the supersaturation point when spontaneous crystallization of the zeolite occurs. Analcime zeolite has also been prepared under high external pressure (200 MPa) at 280 °C for 23 days using sulfur as a directing agent [21].

Recently, a study of the effect of pressure from the autogenous pressure, typically below 2 MPa, to 800 MPa on the hydrothermal synthesis of some aluminosilicate materials has been reported [22]. By applying high external pressures, the crystallization rate of zeolites was accelerated, while an increase in the pressure yielded a product phase change from small pore zeolites with high silicon-based framework density (FD_Si_) to large pore zeolites with lower FD_Si_, i.e., EDI (8R, FD_Si_ = 16.3 T/1000Å^3^) to BPH (12R, FD_Si_ = 14.6 T/1000Å^3^) and LTA (8R, FD_Si_ = 14.2 T/1000Å^3^) to FAU (12R, FD_Si_ = 13.3 T/1000Å^3^). The crystallization of the less dense phase at high pressure is unusual, as the increase in pressure tends to promote the formation of dense phases. This result was explained by the fact that large pore zeolites can accommodate more water molecules in their pores, thus reducing the total volume of the system.

The crystallization of silicalite-1 in fluoride media under 100 MPa has also been reported [23]. The obtained solid presented crystals are around 150 μm, almost 20 times larger than those obtained under autogenous pressure. Interestingly, a high-pressure system seems to inhibit the formation of defects in the framework, being absent any resonance in the ^1^H-^29^Si CP MAS NMR spectrum of the material obtained under 100 MPa. In contrast, resonances corresponding to Q^3^ Si-(OSi)_3_(OH) groups are clearly visible in the spectrum measured for the MFI prepared under autogenous pressure. The effect of external pressure has also been observed on the post-synthesis modification of USY zeolite. The application of high hydrostatic pressures during the so-called surfactant-templating method not only accelerates the mesopore formation but also yields a narrower pore size distribution than standard methods [24]. Although some examples of zeolite synthesis under high pressures can be found in the literature, the effect of the external pressure on the hydrothermal synthesis of SAPO materials has not been reported yet.

SAPO-18 (IZA-SC three-letter code AEI) [6], is a small pore material that has shown high conversion and selectivity to light olefins in the methanol to olefins (MTO) reaction and lower deactivation than the SAPO-34 used in the industrial process [25,26]. Various parameters have been studied to improve the SAPO-18 crystallization and its catalytic properties [27,28,29]. In this work, the hydrothermal synthesis of SAPO-18 under different hydrostatic pressures was explored. The obtained results suggest that the external pressure plays an important role in the crystallization of the SAPO-18 that seems to be related to the effective interaction between the OSDA molecules and inorganic solid, which can be modulated by changing the synthesis mixture concentration.

## 2. Materials and Methods

### 2.1. Apparatus

A homemade warm isostatic press (WIP, see Figure S1) was used as the pressurization apparatus, and deionized water was used as the pressure medium. The maximum treatment temperature and pressure are 150 °C and 200 MPa, respectively. A Teflon^®^ tube purchased from MISUMI was used as the synthesis vessel (see Figure S1). The Teflon^®^ tube was heat-sealed at 330 °C at both ends, which were further sealed with metal clips. The sealed Teflon tube was then placed in the chamber of the WIP.

### 2.2. Synthesis of Silicoaluminophosphates

The silicoaluminophosphates were prepared by following the procedure described previously [25]. The synthesis mixtures had the following composition: 1.6 DIPEA: 0.60 SiO_2_: 1.0 Al_2_O_3_: 0.90 P_2_O_5_: x H_2_O, where x = 9, 25, 50 were hydrothermally treated at 150 °C for a different time. In a typical run, 2.33 g of aluminum hydroxide (Sigma-Aldrich, St. Louis, MO, USA) was added to an aqueous solution of 3.10 g of phosphoric acid (H_3_PO_4_, 85% Wako, Osaka, Japan) in 6.47 g of distilled water. The mixture was stirred at 500 rpm for 1 h using a magnetic stirrer. A second solution containing 3.09 g of *N*,*N*-diisopropylethylamine (DIPEA, Wako), 0.54 g of fumed silica (AEROSIL^®^, Evonik, Essen, Germany) and 5.00 g of distilled water was stirred at 500 rpm for 1 h using a magnetic stirrer. The second mixture was slowly added to the first one and stirred at 500 rpm for 1 h using a magnetic stirrer. The obtained mixture was transferred to Teflon^®^ lined stainless-steel autoclaves and heated at 150 °C under static conditions for 1–72 h when the synthesis was carried out under autogenous pressure (AP). To perform the synthesis under high hydrostatic pressure, the synthesis mixture was sealed in a Teflon^®^ vessel and placed in the Warm Isostatic Press (WIP). The treatment was performed at 150 °C for 1–72 h under different pressures from autogenous pressure to 200 MPa. After the hydrothermal treatment, the solids were recovered by centrifugation, washed with deionized water, and dried. Selected solids were calcined in air at 550 °C for 5 h to remove the OSDA for further characterization.

### 2.3. Characterization

Powder X-ray diffraction patterns were collected in a Rigaku Ultima IV diffractometer (Cu Kα radiation λ = 0.15406 nm, Tokyo, Japan) at 40 kV, 40 mA with a scanning rate of 10° min^−1^. Thermogravimetric analysis was conducted on a Rigaku Thermo plus TG8120 (Tokyo, Japan) from 30 °C to 800 °C with a heating rate of 10 °C min^−1^ with the flowing mixed gas (80 vol% of N_2_ and 20 vol% of O_2_). Chemical analysis was performed on a Thermo Scientific iCAP-6300 inductively (Waltham, MA, USA) coupled plasma atomic emission spectroscopy (ICP-AES). Samples were completely dissolved in a KOH aqueous solution prior to the ICP-AES measurement. ^27^Al and ^31^P magic-angle (MAS) NMR were recorded in a JEOL ECA-300 (Tokyo, Japan) with 300 MHz. Crystal morphology was observed by scanning electron microscope (SEM) using JEOL JSM-7000F. N_2_ adsorption isotherms were measured at −196 °C using a Quantachrome Autosorb-iQ instrument (Boynton Beach, FL, USA).

## 3. Results and Discussion

**Figure 1:**
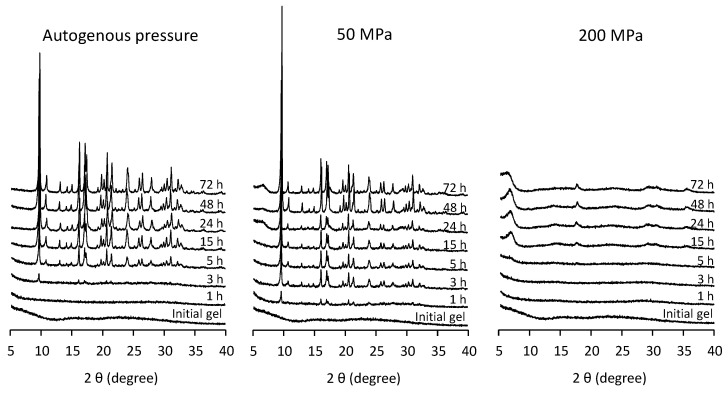
XRD patterns of the obtained solids under autogenous pressure, 50 MPa and 200 MPa at 150 °C for 1 to 72 h using the diluted synthesis mixture (H_2_O/Al_2_O_3_ = 50).

Figure 1 shows the XRD patterns of the obtained solids for different synthesis times at 150 °C under autogenous pressure (AP), 50 and 200 MPa. Diffraction peaks corresponding to SAPO-18 (AEI) can be observed after 3 h of synthesis under autogenous pressure. Increasing the external pressure to 50 MPa allowed us to observe SAPO-18 diffraction peaks after 1 h of synthesis, suggesting the acceleration of the crystallization rate with pressure as was reported in previous work [22]. However, at 200 MPa the SAPO-18 phase crystallization was inhibited, and an unknown phase, possibly a layered material, showing broad diffraction peaks was obtained. The presence of this unknown phase was also confirmed under 50 MPa for synthesis time longer than 24 h.

**Figure 2:**
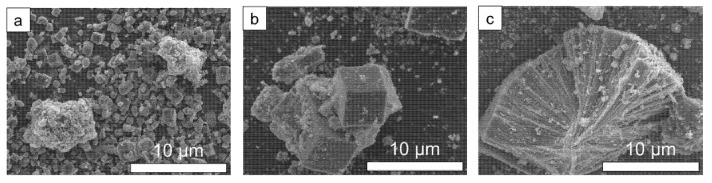
SEM images of the solids obtained under autogenous (**a**), 50 MPa (**b**) and 200 MPa (**c**) at 150 °C for 24 h using the diluted synthesis mixture (H_2_O/Al_2_O_3_ = 50).

SEM images of the solids obtained after 24 h of synthesis are shown in Figure 2. The pure SAPO-18 phase prepared under AP and 50 MPa shows the characteristic cubic crystals. The hydrostatic pressure increase enlarged the crystal size of the SAPO-18 as previously reported for the MFI zeolite [23]. On the other hand, the unknown phase obtained under 200 MPa shows long rod-type layered crystals. Figure S2 shows the SEM images of the solids obtained under AP and 200 MPa at different synthesis times. Cubic crystals started to be observed after 3 h of synthesis under AP and, the crystals continued growing by consuming the initial amorphous solid. On the contrary, all the solids obtained under 200 MPa show amorphous particles similar to those observed in the initial amorphous solid, while the unknown layered crystals start to appear after 5 h of heating, being clearly visible after 15 h.

**Figure 3:**
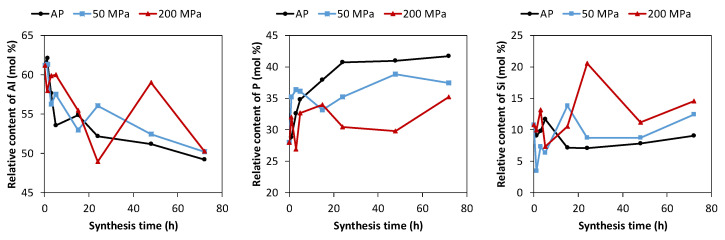
Chemical composition of the solids obtained under autogenous pressure (AP), 50 MPa and 200 MPa at different synthesis time using the diluted synthesis mixture (H_2_O/Al_2_O_3_ = 50).

The relative chemical composition of the solids obtained under autogenous pressure (AP), 50 MPa and 200 MPa at different synthesis times, is shown in Figure 3 and Table S1. The relative Al content of the solids prepared under AP decreases quickly during the first 5 h, from 61% to 54%, showing a value around 50% at long synthesis time, while the relative P content increases in the same time interval, from 28% to 35%; the Si content slightly decreases from 11% to 9%. The main changes in the chemical composition of the solids occur during the nucleation and initial crystal growth steps, showing small changes in the chemical composition after 15 h when the SAPO-18 material is fully crystallized. Crystalline SAPO-18 presents an Al/P ratio higher than 1, while the (Si + P)/Al ratio is close to 1, suggesting the Si atoms are inserted in the framework following mechanism where one P is substituted by one Si (1 P → 1 Si). The samples obtained under 200 MPa shows fluctuations in the chemical composition with time that could be attributed to the presence of amorphous solid unobservable in the XRD patterns. In general, the composition of these solids is more similar to the initial synthesis mixture than the SAPO-18 obtained under AP. Finally, the series of samples prepared under 50 MPa showed an intermediate behavior between the results of the materials obtained under AP and 200 MPa.

**Figure 4:**
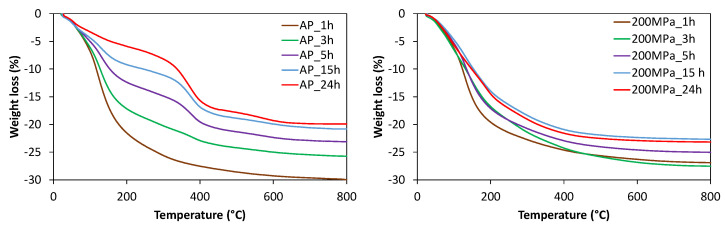
TG profiles of samples obtained under autogenous pressure (AP) and 200 MPa using the diluted synthesis mixtures (H_2_O/Al_2_O_3_ = 50).

Solids obtained under AP and 200 MPa for different times were studied by thermogravimetric analysis. In the samples obtained at short synthesis times, the main weight loss observed in the TG profiles shown in Figure 4, appearing below 250 °C, is assigned to the desorption of occluded water molecules in the solid. The absence of organic molecules is not surprising as at the initial crystallization stage of AlPO materials, the interaction of OSDA molecules with amorphous aluminophosphates is generally weak [30]. A weight loss between 300 and 400 °C is visible in the solids obtained under AP after 3 h, and it increases together with the SAPO-18 crystallinity. These results suggest the OSDA molecules gradually interact with the amorphous precursors acting as OSDA and promoting crystallization. On the other hand, all the solids obtained under 200 MPa presents the main weight loss below 250 °C, suggesting the absence of OSDA molecules incorporated in the unknown phase.

**Figure 5:**
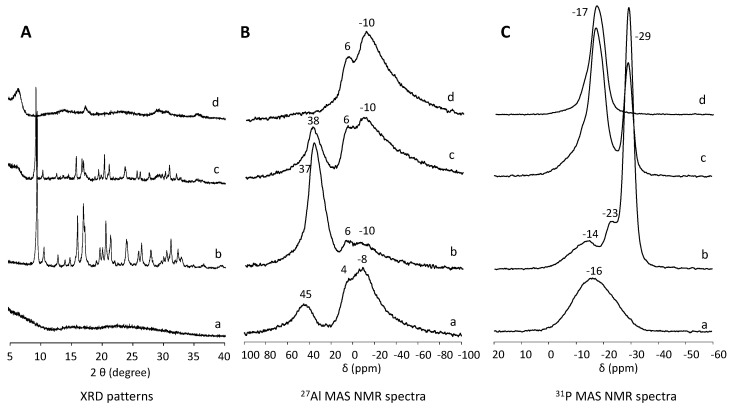
XRD patterns (**A**), and ^27^Al (**B**) and ^31^P (**C**) MAS NMR spectra of the synthesis mixture before heating (a), and as–made solids obtained under autogenous pressure (b), 50 MPa (c) and 200 MPa (d) at 150 °C for 24 h using the diluted synthesis mixture (H_2_O/Al_2_O_3_ = 50).

The chemical environment of the Al and P atoms in the initial stage and the crystalline materials was studied by ^27^Al and ^31^P MAS NMR spectroscopy. Figure 5 shows the XRD patterns and ^27^Al and ^31^P MAS NMR spectra of the initial solid and the products obtained after the hydrothermal synthesis under autogenous pressure, 50 MPa and 200 MPa at 150 °C for 24 h. The initial solid shows an XRD pattern typical of amorphous materials, while in the ^27^Al MAS NMR spectrum, three resonances are observed. The signal centered at 45 ppm is associated with Al atom in tetrahedral coordination in the amorphous aluminophosphate [31], the resonance centered at 4 ppm is attributed to octahedral Al atoms in the unreacted aluminum hydroxide [32]. Lastly, the resonance centered at −8 ppm is assigned to the Al atom in octahedral coordination present in the amorphous aluminophosphate [31]. The ^31^P MAS NMR spectrum shows a broad signal centered at −16 ppm, which is assigned to tetrahedral P atoms present in hydrated aluminophosphates [31]. These results indicate that the initial solid is mainly formed by amorphous silicoaluminophosphate.

Pure SAPO-18 phase was observed in the XRD pattern of the sample obtained under autogenous pressure. The ^27^Al MAS NMR spectrum shows a main resonance centered at 37 ppm assigned to tetrahedral Al atoms accompanied by two minor broad signals centered at 6 and −10 ppm assigned a mixture of penta- and octa-coordinated Al species present in unreacted Al source and amorphous aluminophosphate [32,33]. The ^31^P MAS NMR spectra show the main resonance centered at −29 ppm associated to tetrahedrally coordinated P atoms, a resonance centered at −23 ppm and a broad signal centered at −14 ppm assigned to P atoms which are coordinated to H_2_O molecules forming P(OAl)_x_(H_2_O)_y_, where x = 4 − y, these P species are mainly found at framework defects or on the outer surface of the crystals [33,34,35].

The solid obtained under the highest hydrostatic pressure, 200 MPa, showed a broad diffraction peak centered at 6.7° 2θ (d = 13.2Å). The ^27^Al MAS NMR spectrum of this unknown layered phase shows two resonances centered at 6 and −10 ppm, similar to those observed in the initial amorphous solid, assigned to a mixture of penta- and octa-coordinated Al species present in unreacted Al source and amorphous aluminophosphate. [32,33] In the ^31^P MAS NMR spectrum, one single resonance centered at −17 ppm associated with tetrahedral P atoms is confirmed in hydrated aluminophosphates [31]. This resonance appears a similar chemical shift to that observed in the initial amorphous solid, but the signal is narrower, suggesting the unknown phase is related to the initial aluminophosphate but showing a higher structural order. The XRD pattern, ^27^Al and ^31^P MAS NMR spectra of the solid prepared by hydrothermal synthesis under 50 MPa show a mixture of those obtained for the pure SAPO-18 and the unknown phases.

**Figure 6:**
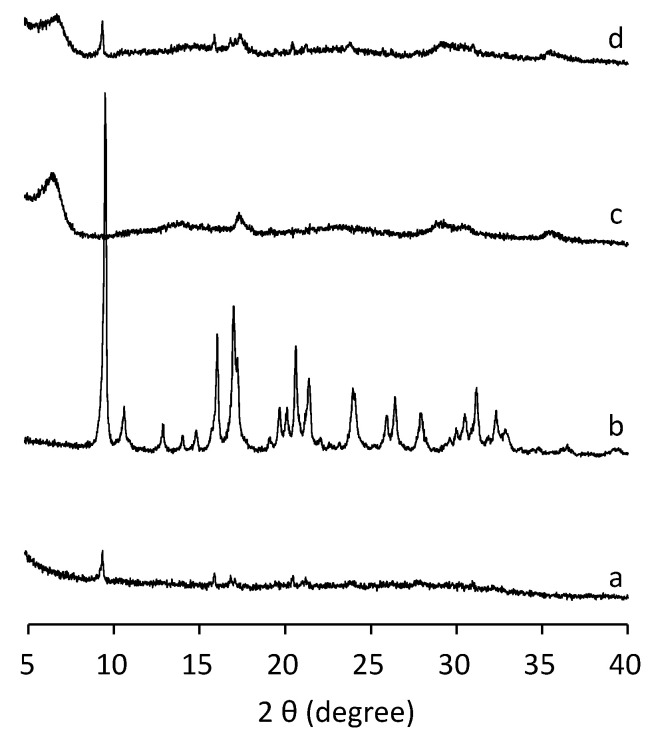
XRD patterns of the solids obtained under autogenous pressure for 3 h (a) and 24 h (b), under 200 MPa for 24 h (c) and under autogenous pressure for 3 h and 200 MPa for 21 h (d) using the diluted synthesis mixture (H_2_O/Al_2_O_3_ = 50).

To study the influence of the pressure in the crystallization of SAPO-18, a two-step synthesis was carried out. First, the synthesis mixture was heated at 150 °C under AP for 3 h. At that time SAPO-18 diffraction peaks were observed. Then, the pressure was raised to 200 MPa, and the synthesis was continued for another 21 h, being the total synthesis time 24 h. A mixture of SAPO-18 and the unknown phase is observed in the XRD pattern as shown in Figure 6, indicating that even in the presence of SAPO-18 crystals, the SAPO-18 phase cannot be obtained under high pressures, but the unknown layered phase crystallizes instead. This result suggests the high hydrostatic pressure not only inhibits the nucleation of the SAPO-18 but also avoids the crystal growth of the SAPO-18 and promotes the formation of the unknown phase.

The synthesis mechanism of AlPO_4_ materials has been studied by several techniques. It was proposed that in the very early stages, the Al and P react, forming amorphous aluminophosphates. The Al-O-P bonds form chains, while the OSDA interacts with them, reducing the interchain electrostatic repulsion and after rearrangements of these chains, the crystalline aluminophosphate structures are formed [36,37]. The organic–inorganic interaction and synthesis mixture concentration play an important role in the formation of microporous aluminophosphates and phase selectivity [38,39].

**Figure 7:**
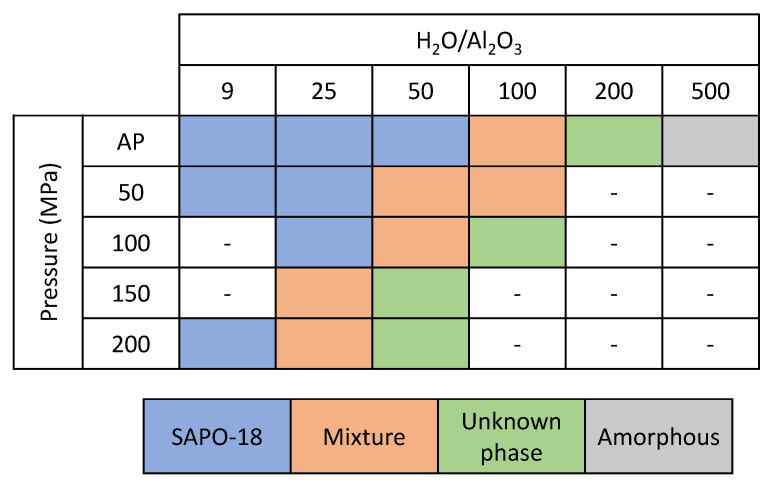
Products obtained with different synthesis mixture concentrations at 150 °C under AP, 50, 100, 150 and 200 MPa of external hydrostatic pressure.

Thus, to promote the organic–inorganic interaction and study the influence of the synthesis mixture concentration under different hydrostatic pressures, the synthesis using lower water content was performed. Figure 7 shows the products obtained at different pressure and H_2_O content, where a clear correlation of the pressure and dilution of the synthesis can be observed. When the amount of water was reduced to an H_2_O/Al_2_O_3_ molar ratio of 25, a pure SAPO-18 phase was obtained under AP and 50 MPa, but a mixture of phases was observed in the synthesis under higher pressures. Lowering the water content until H_2_O/Al_2_O_3_ = 9 allowed to obtain pure SAPO-18 phase under all studied pressures.

Similar to that observed in the diluted system, an increase in hydrostatic synthesis pressure enhanced the crystallization rate of SAPO-18 using an H_2_O/Al_2_O_3_ ratio of 9 (Figure S3). Upon 1 h of hydrothermal treatment, an amorphous XRD pattern was obtained under autogenous pressure, whereas characteristic diffraction peaks of SAPO-18 phase were identified in the solids obtained under 50 and 200 MPa. SAPO-18 solids obtained after 24 h of synthesis under AP, 50 and 200 MPa shows similar XRD patterns. These solids present Al/P and (Si + P)/Al ratios higher than 1 (Table S2), suggesting the Si atoms, in this case, are inserted in the framework following two mechanisms. The first one also observed in the SAPO-18 obtained under AP in diluted concentration synthesis mixtures, where one P is substituted by one Si (1 P → 1 Si) and a second one, where two Si atoms substituted one Al and one P (1 Al + 1 P → 2 Si).

**Figure 8:**
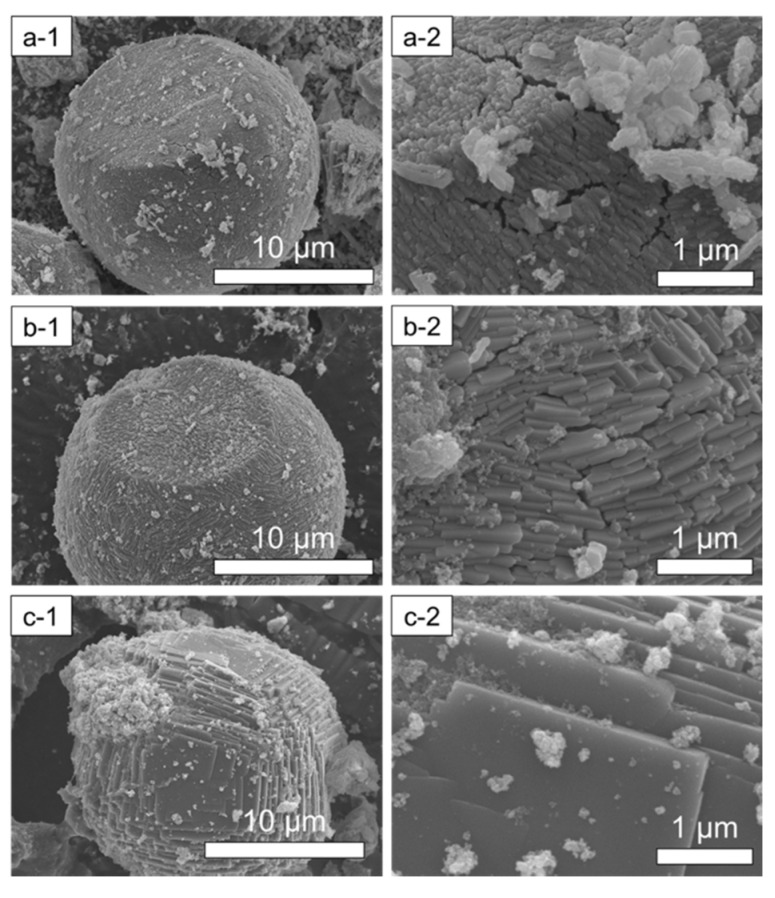
SEM images of SAPO-18 obtained under AP (**a-1**,**a-2**), 50 MPa (**b-1**,**b-2**) and 200 MPa (**c-1**,**c-2**) using the concentrated synthesis mixture (H_2_O/Al_2_O_3_ = 9).

SEM images of SAPO-18 samples obtained under AP, 50 and 200 MPa are shown in Figure 8. All solids present big spheric particles of 17 μm of diameter formed by the agglomeration of small crystals. The size of these cuboid primary crystallites increases with the synthesis pressure, from 0.5 × 0.1 μm in the AP sample, to 4 × 0.5 μm of SAPO-18 obtained under 200 MPa.

The initial synthesis solid (H_2_O/Al_2_O_3_ = 9) shows an XRD pattern typical of amorphous materials and similar ^27^Al and ^31^P MAS NMR spectra than those obtained for the initial synthesis solid prepared with an H_2_O/Al_2_O_3_ = 50. In this case, the SAPO-18 phase was obtained under AP, 50 MPa and 200 MPa, showing similar ^27^Al and ^31^P MAS NMR spectra to SAPO-18 obtained in more diluted conditions (See Figure S4).

Calcined SAPO-18 samples were characterized by N_2_ adsorption to study the influence of the synthesis pressure on the textural properties of the solids (Table 1). SAPO-18 samples obtained under AP and 50 MPa show similar BET surface areas and micropore volumes. The sample obtained under 200 MPa shows lower adsorption capacity at the same time a higher external surface area. This result can be explained by the lower crystallinity of the SAPO-18 and the presence of small particles, probably amorphous solid, as observed in the SEM images.

**Figure 9:**
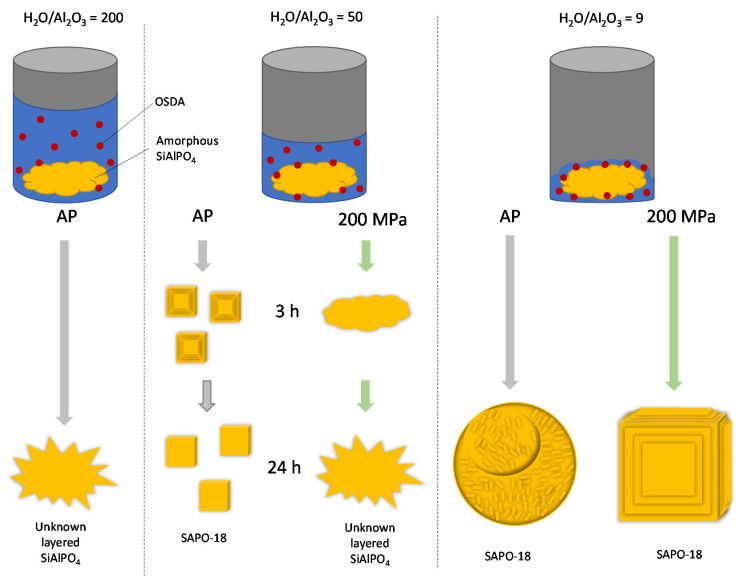
Schematic summary of the most relevant results obtained in the SAPO-18 crystallization under autogenous pressure (AP) and 200 MPa.

Previous results have shown the important effect of the synthesis mixture concentration and hydrostatic pressure on the crystallization and crystal morphology of SAPO-18. Figure 9 shows a schematic summary of the most representative results. When the synthesis was carried out using a typical water content of H_2_O/Al_2_O_3_ = 50 under autogenous pressure, a pure SAPO-18 phase was obtained. At a short synthesis time (3 h), rough surface cubes are observed, suggesting a fast crystal growth promoted by high supersaturation. While at a longer synthesis time (24 h), smooth surface cubes are predominant, indicating a drop in the supersaturation as the amorphous precursors are consumed [40]. On the other hand, the synthesis under 200 MPa resulted in an unknown layered silicoaluminophosphate that did not incorporate any OSDA molecule. This solid was also obtained under autogenous pressure (AP) when the synthesis mixture was diluted to H_2_O/Al_2_O_3_ = 200. The high water content causes a lower ion concentration, reducing the organic-inorganic interaction and the driving force needed for the nucleation and growth of SAPO-18 crystals. When the water concentration was reduced to H_2_O/Al_2_O_3_ = 9, agglomerated crystals forming large spheres of SAPO-18 were obtained under AP and 200 MPa. However, the crystals obtained under AP are very small, suggesting the formation of many nuclei promoted by a high supersaturation. On the other hand, the crystals observed in the sample prepared under 200 MPa show smooth facets characteristics of crystal growth at low supersaturation, similar to that observed at AP when H_2_O/Al_2_O_3_ = 50. These results suggest that under high pressure, the behavior of the ion concentration, and therefore crystal growth, is similar to that observed in a more diluted mixture heated under AP conditions.

## 4. Conclusions

The effect of the external pressure on the hydrothermal synthesis of the microporous material SAPO-18 has been studied. The SAPO-18 phase was obtained by heating a typical synthesis mixture at 150 °C under autogenous pressure (AP). The crystallization of SAPO-18 was speeded up by increasing the hydrostatic pressure to 50 MPa, whereas the synthesis carried out under 200 MPa resulted in the formation of a layered silicoaluminophosphate. The application of high-pressure avoided the incorporation of the OSDA, *N*,*N*-diisopropylethylamine, in the layered silicoaluminophosphate, which could be related to the weakening of the organic-inorganic interaction. The results observed in the two-step synthesis confirm that SAPO-18 crystallization is inhibited under high pressures.

The SAPO-18 phase can be obtained even under 200 MPa by increasing the concentration of the synthesis mixture, which promotes the interaction between the OSDA molecules and the amorphous silicoaluminophosphate. SEM images suggest that the crystal growth mechanism followed under high pressures in concentrated mixtures is more similar to that observed under AP conditions in more diluted mixtures than concentrated ones.

The obtained results suggest that an increase in the synthesis pressure can promote and accelerate the SAPO-18 crystallization. Under the studied conditions, we found that 50 MPa was the optimum hydrostatic pressure that allowed us to obtain pure SAPO-18 in the shortest time.

The effect of pressure on the hydrothermal synthesis of microporous materials has received little attention so far; also, it is frequently studied together with the synthesis temperature. The synthesis procedure showed in this work, allowed us to study the pressure as an independent parameter, opening the use of this synthetic method to understand the crystallization mechanism of microporous materials.

## Figures and Tables

**Table 1:** Textural properties of calcined SAPO-18 samples obtained under autogenous pressure (AP), 50 MPa and 200 MPa using the concentrated synthesis mixture (H_2_O/Al_2_O_3_ = 9).

Sample	BET (m^2^/g) ^a^	A_ext_ (m^2^/g)	V_mic_ (cm^3^/g) ^b^
SAPO-18_AP	508	98	0.17
SAPO-18_50 MPa	493	67	0.17
SAPO-18_200 MPa	431	126	0.12

^a^ calculated using the Brunauer–Emmett–Teller (BET) method. ^b^ determined from the N_2_ adsorption isotherm by applying the *t*-plot method.

## Data Availability

Not applicable.

## Supplementary Materials

The following are available online at https://www.mdpi.com/article/10.3390/nano12030396/s1, Figure S1. Schematic illustration (a) and photo (b) of the home-made warm isostatic press, and photos of the Teflon^®^ reactor (c). Figure S2. SEM images of the initial synthesis gel and solids obtained under autogenous and 200 MPa at 150 °C for different time using the diluted synthesis gel (H_2_O/Al_2_O_3_ = 50). Figure S3. XRD patterns of the obtained solids under autogenous pressure, 50 MPa and 200 MPa at 150 °C for 1 to 72 h using the concentrated synthesis gel (H_2_O/Al_2_O_3_ = 9). Figure S4. XRD patterns (A), and ^27^Al (B) and ^31^P (C) MAS NMR spectra of the synthesis gel before heating (a), and as-made solids obtained under autogenous pressure (b), 50 MPa (c) and 200 MPa (d) at 150 °C for 24 h using the concentrated synthesis gel (H_2_O/Al_2_O_3_ = 9). Table S1. Chemical analysis of the samples obtained at 150 °C for different time using the synthesis gel composition 1.6 DIPEA: 0.60 SiO_2_: 1.0 Al_2_O_3_: 0.90 P_2_O_5_: 50 H_2_O. Table S2. Chemical analysis of the samples obtained at 150 °C for different time using the synthesis gel composition 1.6 DIPEA: 0.60 SiO_2_: 1.0 Al_2_O_3_: 0.90 P_2_O_5_: 9 H_2_O.

## References

[1] Yilmaz B., Müller U. (2009). Catalytic applications of zeolites in chemical industry. Top. Catal..

[2] Li Y., Li L., Yu J. (2017). Applications of Zeolites in Sustainable Chemistry. Chemistry.

[3] Zhang Q., Yu J., Corma A. (2020). Applications of Zeolites to C1 Chemistry: Recent Advances, Challenges, and Opportunities. Adv. Mater..

[4] Wilson S.T., Lok B.M., Messina C.A., Cannan T.R., Flanigen E.M. (1982). Aluminophosphate molecular sieves: A new class of microporous crystalline inorganic solids. J. Am. Chem. Soc..

[5] Zheng C., Li Y., Yu J. (2020). Database of open-framework aluminophosphate structures. Sci. Data.

[6] Database of Zeolite Structures. http://www.iza-structure.org/databases/.

[7] Lok B.M., Messina C.A., Patton R.L., Gajek R.T., Cannan T.R., Flanigen E.M. (1984). Silicoaluminophosphate molecular sieves: Another new class of microporous crystalline inorganic solids. J. Am. Chem. Soc..

[8] Wilson S., Barger P. (1999). The characteristics of SAPO-34 which influence the conversion of methanol to light olefins. Microporous Mesoporous Mater..

[9] Pastore H., Coluccia S., Marchese L. (2005). POROUS ALUMINOPHOSPHATES: From Molecular Sieves to Designed Acid Catalysts. Annu. Rev. Mater. Res..

[10] Martínez-Franco R., Moliner M., Franch C., Kustov A., Corma A. (2012). Rational direct synthesis methodology of very active and hydrothermally stable Cu-SAPO-34 molecular sieves for the SCR of NOx. Appl. Catal. B Environ..

[11] Martínez-Franco R., Moliner M., Yun Y., Sun J., Wan W., Zou X., Corma A. (2013). Synthesis of an extra-large molecular sieve using proton sponges as organic structure-directing agents. Proc. Natl. Acad. Sci. USA.

[12] Li X., Chaikittisilp W., Liu Z., Yanaba Y., Yoshikawa T., Wakihara T., Okubo T. (2015). Synthesis of (Silico)aluminophosphate Molecular Sieves Using an Alkanolamine as a Novel Organic Structure-directing Agent. Chem. Lett..

[13] Cundy C.S., Cox P. (2005). The hydrothermal synthesis of zeolites: Precursors, intermediates and reaction mechanism. Microporous Mesoporous Mater..

[14] Iyoki K., Itabashi K., Okubo T. (2014). Progress in seed-assisted synthesis of zeolites without using organic structure-directing agents. Microporous Mesoporous Mater..

[15] Deguchi M., Iyoki K., Anand C., Yanaba Y., Yoshikawa T., Okubo T., Wakihara T. (2018). Temperature-controlled, two-stage synthesis of ZSM-5 zeolite nanoparticles with Al atoms tetrahedrally coordinated in the framework. Microporous Mesoporous Mater..

[16] Shinno Y., Iyoki K., Ohara K., Yanaba Y., Naraki Y., Okubo T., Wakihara T. (2020). Toward Efficient Synthesis of Chiral Zeolites: A Rational Strategy for Fluoride-Free Synthesis of STW-Type Zeolite. Angew. Chem. Int. Ed..

[17] Ghobarkar H., Schäf O., Paz B., Knauth P. (2003). Zeolite synthesis by simulation of their natural formation conditions: From macroscopic to nanosized crystals. J. Solid State Chem..

[18] Schäf O., Ghobarkar H., Knauth P. (2005). Hydrothermal synthesis of nanomaterials. Nanostructured Materials.

[19] Ghobarkar H., Schäf O., Güth U. (2001). The use of the high pressure hydrothermal method for tailored synthesis of zeolites without structure directing agents. Instance: Synthesis of natural zeolites with 5-1 building units. High Press. Res..

[20] Ghobarkar H., Schäf O., Knauth P. (2001). Zeolite Synthesis by the High-Pressure Hydrothermal Method: Synthesis of Natural 6-Ring Zeolites with Different Void Systems. Angew. Chem. Int. Ed..

[21] Loera S., Lima E., Pfeiffer H., Lara V.H. (2012). Synthesis of aluminosilicates under high pressure and using sulfur as directing agent. Open Chem..

[22] Tan C., Liu Z., Yonezawa Y., Sukenaga S., Ando M., Shibata H., Sasaki Y., Okubo T., Wakihara T. (2020). Unique crystallization behavior in zeolite synthesis under external high pressures. Chem. Commun..

[23] Bergé-Lefranc D., Vagner C., Calaf R., Pizzala H., Denoyel R., Brunet P., Ghobarkar H., Schäf O. (2012). In vitro elimination of protein bound uremic toxin p-cresol by MFI-type zeolites. Microporous Mesoporous Mater..

[24] Sato R., Liu Z., Peng C., Tan C., Hu P., Zhu J., Takemura M., Yonezawa Y., Yamada H., Endo A. (2021). Engineering Mesopore Formation in Hierarchical Zeolites under High Hydrostatic Pressure. Chem. Mater..

[25] Chen J., Thomas J.M., Wright P.A., Townsend R.P. (1994). Silicoaluminophosphate number eighteen (SAPO-18): A new microporous solid acid catalyst. Catal. Lett..

[26] Aguayo A.T., Gayubo A.G., Vivanco R., Olazar M., Bilbao J. (2005). Role of acidity and microporous structure in alternative catalysts for the transformation of methanol into olefins. Appl. Catal. A Gen..

[27] Martínez-Franco R., Li Z., Martínez-Triguero J., Moliner M., Corma A. (2016). Improving the catalytic performance of SAPO-18 for the methanol-to-olefins (MTO) reaction by controlling the Si distribution and crystal size. Catal. Sci. Technol..

[28] Hirota Y., Yamada M., Uchida Y., Sakamoto Y., Yokoi T., Nishiyama N. (2016). Synthesis of SAPO-18 with low acidic strength and its application in conversion of dimethylether to olefins. Microporous Mesoporous Mater..

[29] Knyazeva E.E., Kasnerik V.I., Konnov S.V., Ivanov A.O., Dobryakova I.V., Ivanova I.I. (2018). Effect of Synthesis Temperature Formation of the Structure and Properties of Silicoaluminophosphate with the AEI Structure. Pet. Chem..

[30] Xu J., Chen L., Zeng D., Yang J., Zhang M., Ye C., Deng F. (2007). Crystallization of AlPO4-5 Aluminophosphate Molecular Sieve Prepared in Fluoride Medium: A Multinuclear Solid-State NMR Study. J. Phys. Chem. B.

[31] Agliullin M.R., Faizullin A.V., Khazipova A.N., Kutepov B.I. (2020). Synthesis of Fine-Crystalline SAPO-11 Zeolites and Analysis of Their Physicochemical and Catalytic Properties. Kinet. Catal..

[32] Isobe T., Watanabe T., de la Caillerie J.D., Legrand A., Massiot D. (2003). Solid-state 1H and 27Al NMR studies of amorphous aluminum hydroxides. J. Colloid Interface Sci..

[33] He H., Klinowski J. (1993). Solid-state NMR studies of the aluminophosphate molecular sieve AlPO4-18. J. Phys. Chem..

[34] Buchholz A., Wang W., Xu M., Arnold A., Hunger M. (2002). Thermal stability and dehydroxylation of Brønsted acid sites in silicoaluminophosphates H-SAPO-11, H-SAPO-18, H-SAPO-31, and H-SAPO-34 investigated by multi-nuclear solid-state NMR spectroscopy. Microporous Mesoporous Mater..

[35] Fan D., Tian P., Xu S., Xia Q., Su X., Zhang L., Zhang Y., He Y., Liu Z. (2012). A novel solvothermal approach to synthesize SAPO molecular sieves using organic amines as the solvent and template. J. Mater. Chem..

[36] Fan F., Feng Z., Sun K., Guo M., Guo Q., Song Y., Li W., Li C. (2009). In Situ UV Raman Spectroscopic Study on the Synthesis Mechanism of AlPO-5. Angew. Chem. Int. Ed..

[37] Zhao Z., Xu S., Hu M.Y., Bao X., Hu J.Z. (2016). In Situ High Temperature High Pressure MAS NMR Study on the Crystallization of AlPO4-5. J. Phys. Chem. C.

[38] Zhang B., Xu J., Fan F., Guo Q., Tong X., Yan W., Yu J., Deng F., Li C., Xu R. (2012). Molecular engineering of microporous crystals: (III) The influence of water content on the crystallization of microporous aluminophosphate AlPO4-11. Microporous Mesoporous Mater..

[39] Zhao D., Zhang Y., Li Z., Wang Y., Yu J. (2017). Synthesis of SAPO-18/34 intergrowth zeolites and their enhanced stability for dimethyl ether to olefins. RSC Adv..

[40] Cubillas P., Anderson M.W. (2010). Synthesis Mechanism: Crystal Growth and Nucleation. Zeolites and Catalysis: Synthesis, Reactions and Applications.

