# Supplementary material for: Exploring Hydrothermal Synthesis of SAPO-18 under High Hydrostatic Pressure
Source: Nanomaterials (Basel). 2022 Jan 26;12(3):396. doi: 10.3390/nano12030396 (PMC8838306; doi:10.3390/nano12030396)
Supplement: Supplementary file 1 [file nanomaterials-12-00396-s001.zip › nanomaterials-1526377-supplementary.pdf]

Supplementary Materials

Exploring hydrothermal synthesis of SAPO-18 under high hydrostatic pressure

Exploring Hydrothermal Synthesis of SAPO-18 under High Hydrostatic Pressure

Raquel Simancas [1,†], Masamori Takemura [2,†], Yasuo Yonezawa [1], Sohei Sukenaga [3], Mariko Ando [4], Hiroyuki Shibata [3], Anand Chokkalingam [1], Kenta Iyoki [2], Tatsuya Okubo [2] and Toru Wakihara [1,2,*]

[1] Institute of Engineering Innovation, The University of Tokyo, 2-11-16 Yayoi, Bunkyo-ku, Tokyo 113-8656, Japan; rsimancas@chemsys.t.u-tokyo.ac.jp (R.S.); yone-rice@chemsys.t.u-tokyo.ac.jp (Y.Y.); anand@chemsys.t.u-tokyo.ac.jp (A.C.)

[2] Department of Chemical System Engineering, The University of Tokyo, 7-3-1 Hongo, Bunkyo-ku, Tokyo 13-8656, Japan; takem003@chemsys.t.u-tokyo.ac.jp (M.T.); k_iyoki@chemsys.t.u-tokyo.ac.jp (K.I.); okubo@chemsys.t.u-tokyo.ac.jp (T.O.)

[3] Institute of Multidisciplinary Research for Advanced Materials, Tohoku University, Sendai 980-8577, Japan; sohei.sukenaga.d3@tohoku.ac.jp (S.S.); hiroyuki.shibata.e8@tohoku.ac.jp (H.S.)

[4] Graduate School of Engineering, Tohoku University, Sendai 980-8579, Japan; mariko.ando.b2@tohoku.ac.jp

* Correspondence: wakihara@chemsys.t.u-tokyo.ac.jp

† These authors contributed equally to this work.

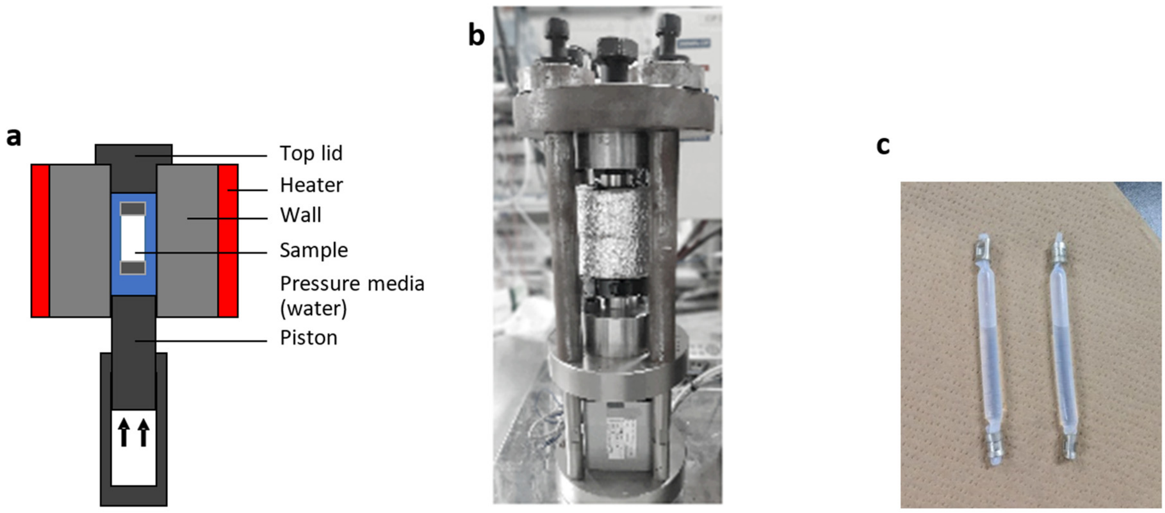

Figure S1. Schematic illustration (**a**) and photo (**b**) of the home-made warm isostatic press, and photos of the Teflon® reactor (**c**).

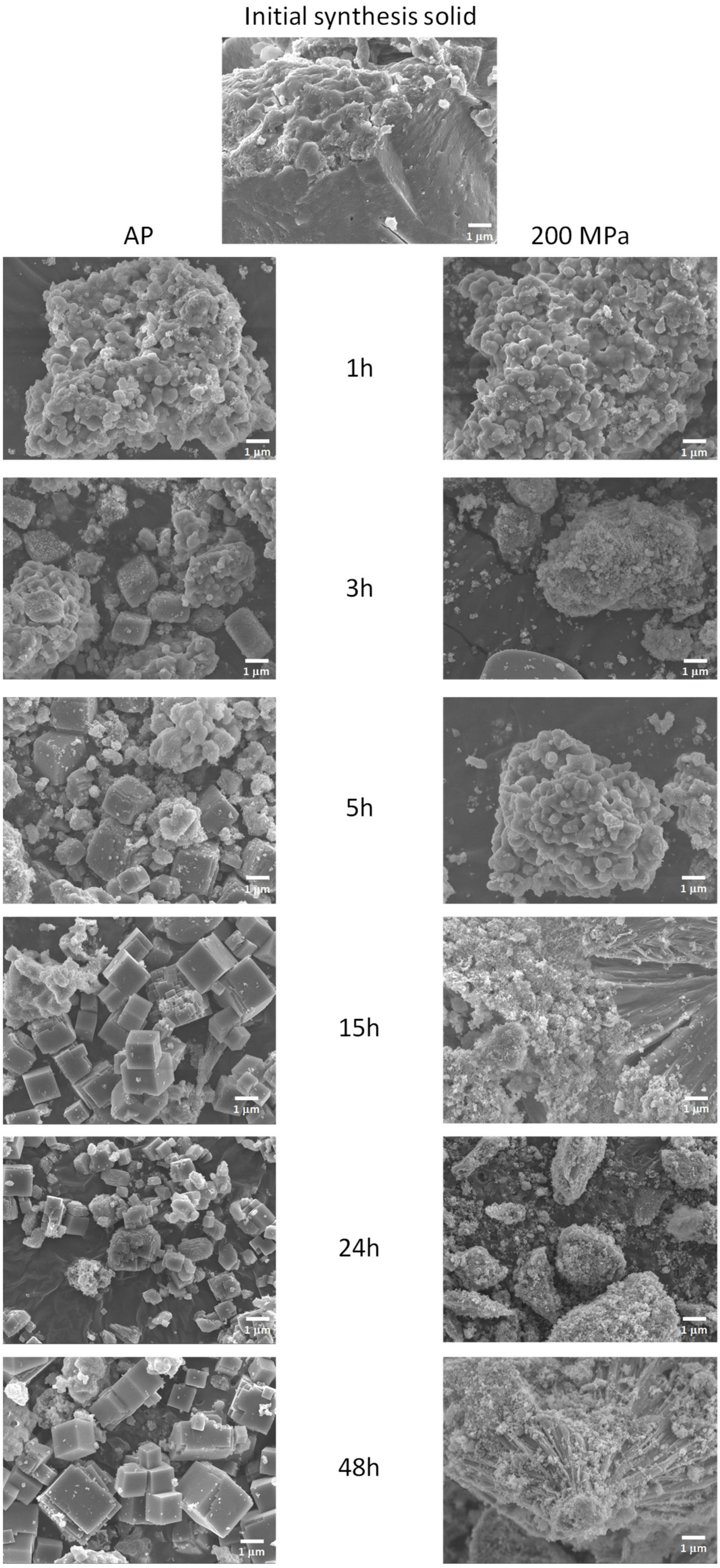

Figure S2. SEM images of the initial synthesis solid and the solids obtained under autogenous (AP) and 200 MPa at 150°C for different time using the diluted synthesis mixture (H_2O/Al_2O_3=50).

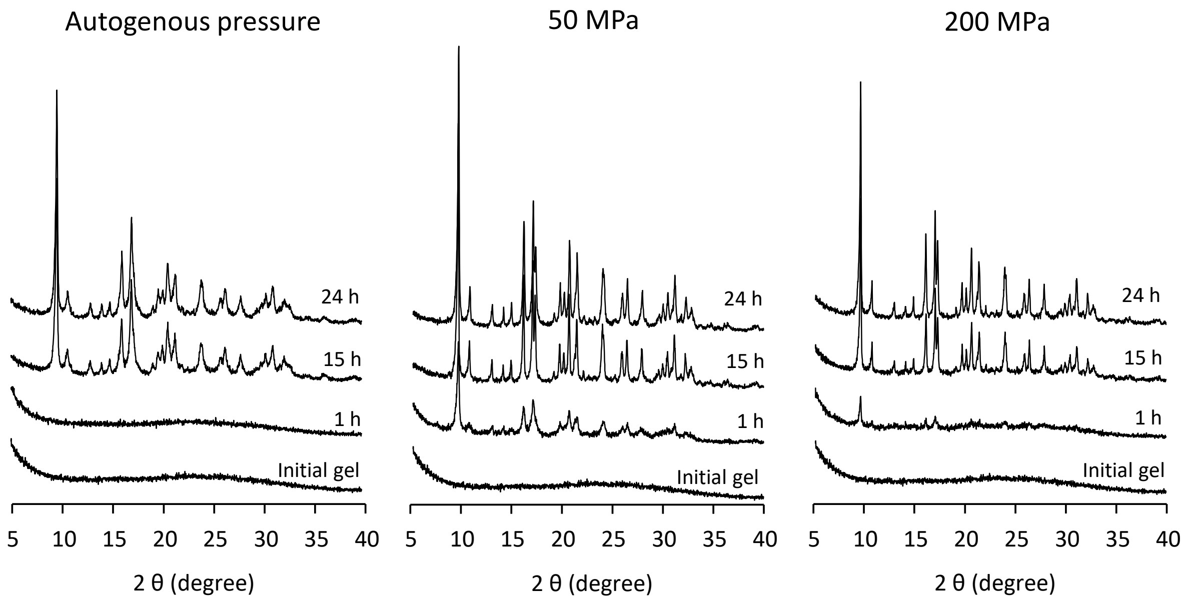

Figure S3. XRD patterns of the obtained solids under autogenous pressure, 50 MPa and 200 MPa at 150 °C for 1 to 72 h using the concentrated synthesis mixture (H_2O/Al_2O_3=9).

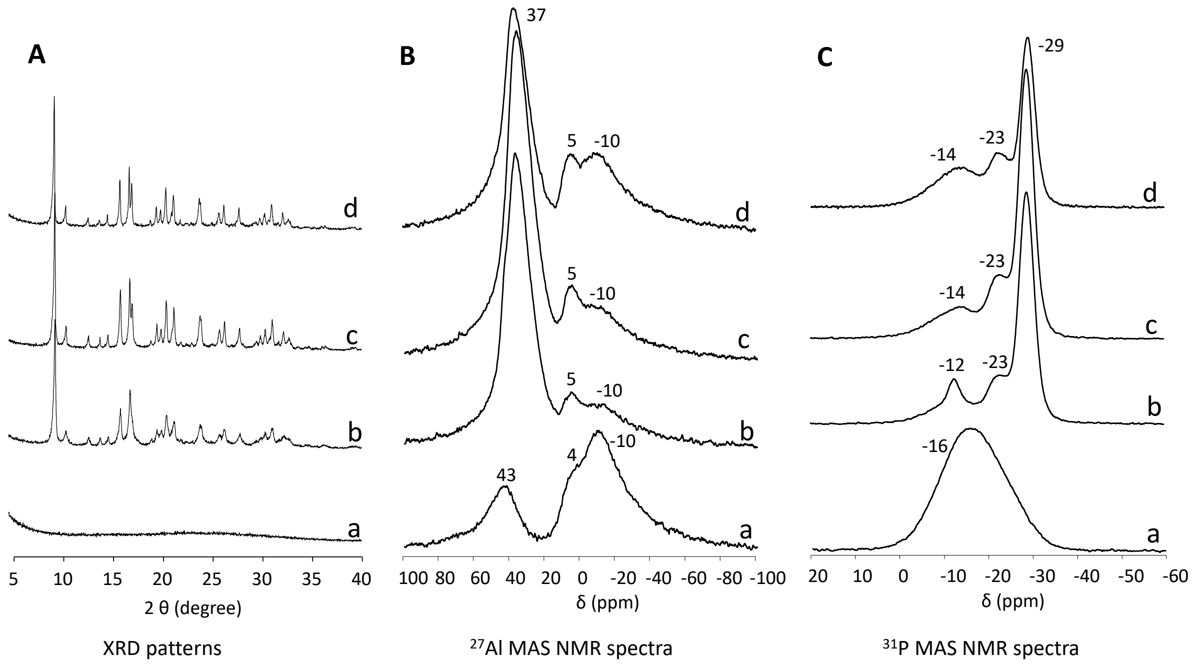

Figure S4. XRD patterns (**A**), and ^{27}Al (**B**) and ^{31}P (**C**) MAS NMR spectra of the synthesis mixture before heating (a), and as-made solids obtained under autogenous pressure (b), 50 MPa (c) and 200 MPa (d) at 150°C for 24 h using the concentrated synthesis mixture (H_2O/Al_2O_3=9).

Table S1. Chemical analysis of the samples obtained at 150°C for different time using the synthesis mixture composition 1.6 DIPEA : 0.60 SiO_2 : 1.0 Al_2O_3 : 0.90 P_2O_5 : 50 H_2O.

**Sample**	**Al (mol%)**	**P (mol%)**	**Si (mol%)**	**Al/P**	**Si/(Si+Al+P)**	**(Si+P)/Al**
Initial gel	61	28	11	2.2	0.11	0.63
AP 1h	62	29	9	2.2	0.09	0.61
AP 3h	58	33	10	1.8	0.10	0.73
AP 5h	54	35	12	1.5	0.12	0.87
AP 15h	55	38	7	1.5	0.07	0.82
AP 1d	52	41	7	1.3	0.07	0.92
AP 2d	51	41	8	1.3	0.08	0.95
AP 3d	49	42	9	1.2	0.09	1.03
50MPa 1h	61	35	4	1.7	0.03	0.63
50MPa 3h	56	36	7	1.6	0.07	0.78
50MPa 5h	58	36	6	1.6	0.06	0.74
50MPa 15h	53	33	14	1.6	0.14	0.89
50MPa 1d	56	35	9	1.6	0.09	0.78
50MPa 2d	52	39	9	1.4	0.09	0.91
50MPa 3d	50	37	12	1.3	0.12	0.73
200MPa 1h	58	32	10	1.8	0.10	0.67
200MPa 3h	60	27	13	2.2	0.13	0.67
200MPa 5h	60	33	7	1.8	0.07	0.80
200MPa 15h	56	34	11	1.6	0.11	1.04
200MPa 1d	49	31	21	1.6	0.21	0.69
200MPa 2d	59	30	11	2.0	0.11	0.99
200MPa 3d	50	35	15	1.4	0.15	0.73

Table S2. Chemical analysis of the samples obtained at 150°C for different time using the synthesis mixture composition 1.6 DIPEA : 0.60 SiO_2 : 1.0 Al_2O_3 : 0.90 P_2O_5 : 9 H_2O.

Sample	Al (mol%)	P (mol%)	Si (mol%)	Al/P	Si/(Si+Al+P)	(Si+P)/Al
Initial gel	50	34	16	1.5	0.16	0.99
AP 1h	54	30	16	1.8	0.16	0.85
AP 15h	48	42	11	1.1	0.11	1.10
AP_1d	46	42	12	1.1	0.12	1.16
50MPa 1h	64	33	3	1.9	0.03	0.57
50MPa 15h	50	43	7	1.2	0.07	1.01
50MPa_1d	49	42	9	1.2	0.09	1.06
200MPa 1h	53	33	15	1.6	0.15	0.90
200MPa 15h	49	39	12	1.3	0.12	1.05
200MPa_1d	49	39	13	1.2	0.13	1.06
